# Supplementary material for: Predicting road quality using high resolution satellite imagery: A transfer learning approach
Source: PLoS One. 2021 Jul 9;16(7):e0253370. doi: 10.1371/journal.pone.0253370 (PMC8270213; doi:10.1371/journal.pone.0253370)
Supplement: S1 Appendix — (PDF) [file pone.0253370.s001.pdf]

Technical innovation in the use of CNNs to analyze satellite imagery

Fully-connected layers

In stark contrast to most econometric models, state-of-the-art CNNs contain hundreds of millions of parameters [1]. Most of these parameters come from what are called fully-connected layers, that host a large number of neurons. In AlexNet, for example, the first and the second fully-connected layers, which have 4096 neurons each, contain around 89% of all parameters [2]. A larger number of parameters leads to higher memory requirements when mapping and running the network on computational platforms. Consequently, using deep CNNs obligates significant hardware resources, which hinders their practicability for mobile computing devices that have limited memory specifications [3]. Thus, reducing the parameters of the fully-connected layers in CNNs is important to enable CNN model convergence on relatively limited training data. Li, Fu, et al. added dropout and a Restricted Boltzmann Machine (RBM) to the fully-connected layers, which reduced the number of parameters [4]. Li used the UC Merced land use dataset to evaluate the performance of this method and achieved better performance than other related methods, reaching an overall accuracy of 95.11%. Zhong, Fei, and Zhang proposed a global average pooling layer (GAP), which was used to replace a fully-connected network as the classifier. It greatly reduced the total parameters in a large patch convolutional neural network (LPCNN), and made it easier to train the model with limited training data [5]. The LPCNN was evaluated on three different high spacial resolution remote-sensing datasets: IKONOS land use dataset, UC Merced land use dataset, and Google Earth land use dataset of SIRI-WHU [6–8]. Their improved CNN model achieved the best performance on the three datasets with overall accuracies of 92.54%, 89.90%, and 89.88%, respectively.

Classifiers

In computer vision, logistic, ReLU, hyperbolic tangent (tanh), softmax, and SVM classifiers are commonly used in the output layer of a CNN to divide the feature space and obtain classification results. In remote sensing, a softmax classifier is sometimes inadequate for dividing the feature space since the training data may contain relatively high noise levels, or large differences in appearance (such as in scale) may exist for a particular feature [9]. A few studies have aimed to find the optimal classifier for their specific RS image classification task. In one example, to address the problem of speckle noises in synthetic aperture radar (SAR) images used to detect ships, Bai, Jiang, Pei, Zhang, and Bai substituted a fuzzy SVM for a conventional classifier, thereby reducing the impact of noise sample points on the division of the feature space [10]. As a result, they achieved a detection accuracy of 98.6% for identifying ships in the SAR images. In another example, Xu et al. established a multi-kernel SVM classifier using a linear combination of multiple kernel functions based on the UC Merced land use dataset and the WHU-RS19 scene classification dataset [7, 11, 12]. This classifier adaptively selects a kernel function for classification based on differences within an image feature and, consequently, has a greater ability to divide complex feature spaces, resulting in higher classification accuracy [9].

Loss functions

Like images produced from close-range cameras, RS images produced using aerial or satellite photographic techniques from above are multidirectional, meaning objects of the same type can possess more than one appearance depending on the capture angle. Some commonly used loss functions may not be effective at differentiating features of objects within the same class due to this multidirectionality [9]. There are a few studies that have focused on improving loss functions in this regard. An example, Cheng, Zhou, and Han added L2 regularization and a regularization constraint term that restricted the rotation variation in objects to the original loss function [13]. They evaluated the performance of this method with the NWPU VHR-10 object detection dataset, which includes aircraft, ships, bridges, and so on, improving classification accuracy on 9 of 10 classes versus without the rotation-invariant method. Li, Qu, and Peng designed a loss function in which the intraclass compactness and interclass separability are maximized simultaneously for ship detection in SAR images [14]. In their study, they designed a dense residual CNN based on ResNet50 and used the OpenSARShip dataset to evaluate ship types [15, 16], achieving a 77.2% classification accuracy, which was higher than that of the original ResNet50 for this application.

Network structure

To an extent, increasing the number of layers in neural networks is sometimes associated with improved generalization [9]. However, the increase in the number of network layers means an increase in the number of model parameters to be trained, which generally requires more training data or the use of other techniques to reduce the chance of overfitting [17].

Several studies have sought to improve the network structure of CNNs in remote sensing. A common idea is to design several independent CNN models that differ in convolutional layer depth or the number of neurons in the fully-connected layers, and then combine them through feature fusion or model integration. This idea limits the total number of network parameters and allows the network to converge with limited training data [9]. For example, in order to extract the objects in a built-up area from SAR images, Li, Zhang, and Li extracted features at three different scales using three independent CNN models that differed in the depth of the convolutional layers [18]. They then imported the extracted features of the various scales into the fully-connected layers to fuse them, and then finally classified the fused features using a softmax. This network structure was capable of learning detailed and abstract features in buildings in an urban area, improving the learning ability compared to any of the independent CNNs. Using the UC Merced land use scene classification dataset, Li, Fu, et al. first trained several independent CNN models that differed in the number of neurons in the fully-connected layers and then integrated the classification results using a voting strategy during the test stage to obtain the final scene classification results [4]. Their experimental results (92.14% accuracy) showed that this structure could yield a more accurate scene classification.

Parameter optimization

Training a deep CNN architecture from scratch with random initialization can be computationally prohibitive for large amounts of data, therefore, parameter initialization in CNN remote sensing is generally developed from pre-trained CNN models (e.g., AlexNet and VGG-Net) [9,19]. Pre-trained networks allow practitioners to rapidly transfer and apply the learned parameter values from random image features to their applications, thereby making training for RS image classification more efficient, reducing the complexity and cost of training [9]. The process framework for transfer learning is to use a model trained in one source domain or task to help build a model in a related target domain without the need for considerable new labeled data. This knowledge transfer paradigm, in which general features learned from one task help inform a similar task, has become particularly popular with deep CNNs as pre-trained models built on large labeled datasets such as ImageNet are often available through open source code repositories [20]. While complex representations at later layers can reduce the performance of transfer learning to new tasks if they are too highly specialized, research suggests that transferring features, even from dissimilar tasks, can be better than using random parameter initializations [21]. Transfer learning in the context of using deep CNNs on satellite images has recently shown to be effective for general object classification, residential scene classification, and to predict locations of conflict-related deaths [19, 22, 23]. According to the literature, there are currently two commonly employed transfer learning approaches to initialize parameters for CNN training in RS image classification [24–26]. One of the approaches selects several layers of a pre-trained network and fine-tunes them based on the remote sensing image dataset, so that the CNN adapts to achieve improved RS image generalization. For example, Zhang, Wang, Liu, Liu, and Wang compiled a remote sensing image set of five types of urban land—commercial, residential, factory, education-purposed, and public land, based on Google Earth satellite images [27]. They then trained a prediction model by fine-tuning a pre-trained AlexNet using this sample set. Subsequently, they used the prediction model to classify images into the five types of urban land in the cities of Shenyang and Beijing. Their results demonstrated that the fine-tuned pre-trained AlexNet could effectively classify urban functional land. The second transfer learning approach directly uses a pre-trained network as an extractor for obtaining remote sensing image features, with the extracted features then used to train a classifier [9]. For example, Weng, Mao, Lin, and Guo used the last convolutional layer of a pre-trained AlexNet network to extract remote sensing image features to train an extreme learning machine classifier [28]. This classifier achieved a classification accuracy of 95.62% on the UC Merced land use dataset. Marmanis, Datcu, Esch, and Stilla converted 1-D remote sensing image features extracted by the fully-connected layers of a pre-trained network to a 2-D feature matrix [29]. This matrix was then used to train a CNN model containing two convolutional layers, two fully-connected layers, and a softmax classifier. They then used their model to classify the scenes in the UC Merced land use dataset, achieving an overall classification accuracy of 92.4%. Lu et al. used the network parameters obtained from training a linear land elimination task as the initial parameters to train a CNN, subsequently using the eigenvectors from the trained CNN model as the input for a SVM to identify farmland from UAV images [30].

Parameter optimization is also a key component of the training process, i.e. updating CNN weights, specified by a learning optimizer. Popular training optimizers include stochastic gradient descent (SGD), root mean square propagation (RMSprop), Adam, Adadelta, and Adagrad. As one example, in their use of a large patch CNN for land use scene classification, Zhong et al. employed SGD as an optimizer, and a momentum technique is used to help prevent the model from getting stuck in local minima and to speed the approach to the global minimum [5].

Application cases of CNN-based RS image classification are commonly divided into scene classification [11, 22], object detection [10,31], object extraction [32–34], object classification [10,19], and object qualification [35]—broadly the concern of the present work. Scene classification is a task in which the entirety of an RS image is classified using the overall context and information contained within the image [9]. Object detection is the process of determining the locations and types of targets to be detected in a remote sensing image and labeling their locations and types with bounding boxes [9]. Object extraction is the process of determining the accurate boundaries of the objects to be extracted in a remote sensing image [9]. Object classification is the determination of what an object of interest is.

References

[1] Simonyan K, Zisserman A. Very deep convolutional networks for large-scale image recognition. arXiv preprint arXiv:14091556. 2014;.

[2] Krizhevsky A, Sutskever I, Hinton GE. Imagenet classification with deep convolutional neural networks. Communications of the ACM. 2017;60(6):84–90.

[3] Zhou H, Alvarez JM, Porikli F. Less Is More: Towards Compact CNNs. In: Leibe B, Matas J, Sebe N, Welling M, editors. Computer Vision – ECCV 2016. Cham: Springer International Publishing; 2016. p. 662–677.

[4] Li H, Fu K, Xu G, Zheng X, Ren W, Sun X. Scene classification in remote sensing images using a two-stage neural network ensemble model. Remote Sensing Letters. 2017;8(6):557–566.

[5] Zhong Y, Fei F, Zhang L. Large patch convolutional neural networks for the scene classification of high spatial resolution imagery. Journal of Applied Remote Sensing. 2016;10(2):025006.

[6] Govt U. IKONOS-2 - CKAN; 2020. `https://catalog.data.gov/dataset/ikonos-2`.

[7] UofC. UC Merced Land Use Dataset; 2010. `http://weegee.vision.ucmerced.edu/datasets/landuse.html`.

[8] LLC G. SIRI_WHU Dataset; 2019. *figshare* `https://figshare.com/articles/dataset/SIRI_WHU_Dataset/8796980`.

[9] Song J, Gao S, Zhu Y, Ma C. A survey of remote sensing image classification based on CNNs. Big Earth Data. 2019;3(3):232–254. doi:10.1080/20964471.2019.1657720.

[10] Bai Y, et al. Application of animproved ELU convolution neural network in the SAR image ship detection. Bull Surv Mapping. 2018;.

[11] Suhui X, Xiaodong M, Peng Z, Ji M. Scene classification of remote sensing image based on multi-scale feature and deep neural network. Acta Geodaetica et Cartographica Sinica. 2016;45(7):834.

[12] University W. 19-Class Satellite Scene Dataset; 2018. *figshare* `https://figshare.com/articles/dataset/Untitled_Item/6086159#__sid=js0`.

[13] Cheng G, Zhou P, Han J. Learning rotation-invariant convolutional neural networks for object detection in VHR optical remote sensing images. IEEE Transactions on Geoscience and Remote Sensing. 2016;54(12):7405–7415.

[14] Li J, Qu C, Peng S. Ship classification for unbalanced SAR dataset based on convolutional neural network. Journal of Applied Remote Sensing. 2018;12(3):035010.

[15] He K, Zhang X, Ren S, Sun J. Deep residual learning for image recognition. In: Proceedings of the IEEE conference on computer vision and pattern recognition; 2016. p. 770–778.

[16] Huang L, Liu B, Li B, Guo W, Yu W, Zhang Z, et al. OpenSARShip: A dataset dedicated to Sentinel-1 ship interpretation. IEEE Journal of Selected Topics in Applied Earth Observations and Remote Sensing. 2017;11(1):195–208.

[17] Abu-Mostafa ea Yaser S. Learning from Data. AMLBook; 2012.

[18] Li J, Zhang R, Li Y. Multiscale convolutional neural network for the detection of built-up areas in high-resolution SAR images. In: 2016 IEEE International Geoscience and Remote Sensing Symposium (IGARSS). IEEE; 2016. p. 910–913.

[19] Chew RF, Amer S, Jones K, Unangst J, Cajka J, Allpress J, et al. Residential scene classification for gridded population sampling in developing countries using deep convolutional neural networks on satellite imagery. Int J Health Geogr. 2018;17:12. doi:10.1186/s12942-018-0132-1.

[20] Stanford Vision Lab PU Stanford University. ImageNet; 2020. http://www.image-net.org/.

[21] Yosinski J, Clune J, Bengio Y, Lipson H. How transferable are features in deep neural networks? In: Ghahramani Z, Welling M, Cortes C, Lawrence ND, Weinberger KQ, editors. Advances in Neural Information Processing Systems 27. Curran Associates, Inc.; 2014. p. 3320–3328. Available from: http://papers.nips.cc/paper/5347-how-transferable-are-features-in-deep-neural-networks.pdf.

[22] Goodman S, BenYishay A, Runfola D. A convolutional neural network approach to predict non-permissive environments from moderate-resolution imagery. Transactions in GIS. 2020;doi:10.1111/tgis.12661.

[23] Marmanis D, Datcu M, Esch T, Stilla U. Deep Learning Earth Observation Classification Using ImageNet Pretrained Networks. IEEE GEOSCIENCE AND REMOTE SENSING LETTERS. 2016;13(1). doi:10.1109/LGRS.2015.2499239.

[24] Zhou W, Shao Z, Cheng Q. Deep feature representations for high-resolution remote sensing scene classification. In: 2016 4th International Workshop on Earth Observation and Remote Sensing Applications (EORSA). IEEE; 2016. p. 338–342.

[25] Castelluccio M, Poggi G, Sansone C, Verdoliva L. Land use classification in remote sensing images by convolutional neural networks. arXiv preprint arXiv:150800092. 2015;.

[26] Nogueira K, Penatti OA, Dos Santos JA. Towards better exploiting convolutional neural networks for remote sensing scene classification. Pattern Recognition. 2017;61:539–556.

[27] Zhang Q, Wang Y, Liu Q, Liu X, Wang W. CNN based suburban building detection using monocular high resolution Google Earth images. In: 2016 IEEE International Geoscience and Remote Sensing Symposium (IGARSS). IEEE; 2016. p. 661–664.

[28] Weng Q, Mao Z, Lin J, Guo W. Land-use classification via extreme learning classifier based on deep convolutional features. IEEE Geoscience and Remote Sensing Letters. 2017;14(5):704–708.

[29] Marmanis D, Datcu M, Esch T, Stilla U. Deep learning earth observation classification using ImageNet pretrained networks. IEEE Geoscience and Remote Sensing Letters. 2015;13(1):105–109.

[30] Lu H, Fu X, He Y, Li L, Zhuang W, Liu T. Cultivated land information extraction from high resolution UAV images based on transfer learning. Transactions of the Chinese Society of Agricultural Machinery. 2015;46:274–279.

[31] Zhang T, Zhang X. High-Speed Ship Detection in SAR Images Based on a Grid Convolutional Neural Network. Remote Sensing. 2019;11(10):1206. doi:10.3390/rs11101206.

[32] Zhang Y, Xia W, Zhang YZ, Sun SK, Sang LZ. Road extraction from multi-source high-resolution remote sensing image using convolutional neural network. In: 2018 International Conference on Audio, Language and Image Processing (ICALIP). IEEE; 2018. p. 201–204.

[33] Cheng G, Wang Y, Xu S, Wang H, Xiang S, Pan C. Automatic Road Detection and Centerline Extraction via Cascaded End-to-End Convolutional Neural Network. IEEE Transactions on Geoscience and Remote Sensing. 2017;55(6):3322–3337. doi:10.1109/TGRS.2017.2669341.

[34] Xu Y, Wu L, Xie Z, Chen Z. Building extraction in very high resolution remote sensing imagery using deep learning and guided filters. Remote Sensing. 2018;10(1):144.

[35] Cadamuro G, Muhebwa A, Taneja J. Assigning a Grade: Accurate Measurement of Road Quality Using Satellite Imagery. CoRR. 2018;abs/1812.01699.
