# Supplementary material for: Predicting road quality using high resolution satellite imagery: A transfer learning approach
Source: PLoS One. 2021 Jul 9;16(7):e0253370. doi: 10.1371/journal.pone.0253370 (PMC8270213; doi:10.1371/journal.pone.0253370)
Supplement: Attachment — Submitted filename: Response to Reviewers.pdf [file pone.0253370.s002.pdf]

Answers to Editorial Comments

Comment EC.1 *Please ensure that your manuscript meets PLOS ONE's style requirements, including those for file naming.*
Answer to EC.1 Thank you for the opportunity to update our style to match PLOS ONE. We believe we have corrected any remaining issues. The specific changes we made are:

- Changed figure and table labels to exactly match filenames and in-text references.
- Included initials in parenthesis for the corresponding author email.
- Rewrote section headings in sentence form.

Comment EC.2 *Please review your reference list to ensure that it is complete and correct.*
Answer to EC.2 This review has been completed; as a part of this review, we have also added a number of citations to improve our coverage of the literature.

Comment EC.3 *Please ensure that you refer to Figure 3 in your text as, if accepted, production will need this reference to link the reader to the figure.*
Answer to EC.3 Fig 3 has been removed in response to editorial comment 5.

Comment EC.4 *We note you have included a table to which you do not refer in the text of your manuscript. Please ensure that you refer to Table 3 in your text; if accepted, production will need this reference to link the reader to the Table.*
Answer to EC.4 Table 3 (now Table 4) has now been referenced in the paragraph that precedes it.

Comment EC.5 *We note that Figures 2, 3, 6, 7 and 8 in your submission contain satellite images which may be copyrighted. All PLOS content is published under the Creative Commons Attribution License (CC BY 4.0), which means that the manuscript, images, and Supporting Information files will be freely available online, and any third party is permitted to access, download, copy, distribute, and use these materials in any way, even commercially, with proper attribution. For these reasons, we cannot publish previously copyrighted maps or satellite images created using proprietary data, such as Google software (Google Maps, Street View, and Earth).*
Answer to EC.5 Permission has been granted for use of the state of Virginia's imagery, which is public domain with a request for attribution. The two figures featuring Google Maps images have been removed with some of the caption content transferred to the body of the article.

Answers to Reviewer 1

Comment R1.1 *Author should add a little background of the study and limitations of the existing works and clearly explain the contributions at the end of the introduction.*
Answer to R1.1 Thank you for your comments - they are all appreciated, and we believe have resulted in a better piece. In response to this comment, we have added about two paragraphs of content to our section on Related works, focusing on ensuring the limitations of existing work are clear and our contributions are explicitly described:

"In 2018, Oshri et al. used two data sources in a supervised learning setting: survey data from Afrobarometer [48] as ground truth infrastructure quality labels, and relatively low resolution (10 and 30 meter) satellite imagery from Landsat 8 and Sentinel 1 as input sources to classify, among other infrastructure items, road quality in a binary fashion [49]. They achieved 70.5% accuracy. Also in 2018, Gabriel Cadamuro et al. carried out work utilizing CNNs to classify road quality from satellite images [17]. Here, the International Roughness Index (IRI) was collected by specialized equipment for over 7,000 km of predominantly trunk roadways in Kenya. Using the IRI information to label 50x50 cm resolution satellite images of the corresponding roads, the pre-trained networks AlexNet, VGG, and SqueezeNet were used to classify road segments, yielding accuracy scores shown in Table 1 for binary and 5-category classification. Cadamuro et al. described two key remaining challenges to road classification: (1) treating the problem as sequential, i.e., for the prediction of a given road segment, utilize the data of nearby road segments, and (2) better accommodation for the continuous nature of road roughness measurements to mitigate the negative impact of road heterogeneity on the quality of predictions [17]. This paper contributes to this growing body of literature in a number of ways, including tests incorporating higher resolution imagery, exploration of the effectiveness of intercontinental transfer learning, and the implementation of a continuous measurement of road roughness."

Comment R1.2 *All the key terms of the equations must be mentioned.*
Answer to R1.2 We have carefully checked equations 1 and 2 to ensure that all of our notation is defined in either the body preceding or immediately after each equation. We are confident everything is well defined, but would welcome anything specific we have missed (and apologies in advance if we haven't caught something!)

Comment R1.3 *Reorganize the introduction, trying to explain every word of the title.*
Answer to R1.3 This was a particularly helpful comment - in our short introduction we now try to more explicitly describe what this paper seeks to accomplish, and how that inter-relates with the title:

"Predicting road quality using remote, automated analysis of high resolution images collected from aerial or satellite platforms provide a globally systematic solution to this challenge. In this paper, we present a test of the use of transfer learning in convolutional neural networks in conjunction with high resolution satellite imagery of roads to determine (a) if road quality can be estimated with a reasonable degree of accuracy with satellite imagery, and (b) the degree to which such an approach can be applied across different geographies. Our paper is structured as follows..."

Comment R1.4 *I can see some paragraph in introduction, related work and proposed approach which should be merged. Please reduce text and improve the representation of this section.*
Answer to R1.4 Based on both this and the second comment of the second reviewer, the original

Introduction has been split into an Introduction section and a Related works section. We believe this more focused structure helps the paper read significantly better, and thank the reviewers for their suggestions.

Comment R1.5 *Relevant literature review of latest similar research studies on the topic at hand must be discussed.*
Answer to R1.5 We have added a more detailed discussion of the two papers in recent literature that have engaged with this topic (Oshri et al 2018 and Cadamuro et al. 2018), as well as added a table illustrating the most recently reported accuracy results from Cadumuro's research (our new Table 1). This discussion is also highlighted in our response to R1.1.

Comment R1.6 *Authors should add the most recent reference: 1) Anomaly Detection in Automated Vehicles Using Multistage Attention-Based Convolutional Neural Network, IEEE Transactions on Intelligent Transportation Systems 2) CANintelliIDS: Detecting In-Vehicle Intrusion Attacks on a Controller Area Network using CNN and Attention-based GRU, IEEE Transactions on Network Science and Engineering*
Answer to R1.6 Citations for these two papers have been added to the first sentence of Related works.

Answers to Reviewer 2

Comment R2.1 *What are the main contributions of the current work?*
Answer to R2.1 Firstly, thank you for your comments - both these and reviewer 1's questions helped us produce a much better structured piece. In direct response to this question, we now explicitly discuss the most recent questions highlighted by the literature, and how we respond and engage with those:

"Cadamuro et al. described two key remaining challenges to road classification: (1) treating the problem as sequential, i.e., for the prediction of a given road segment, utilize the data of nearby road segments, and (2) better accommodation for the continuous nature of road roughness measurements to mitigate the negative impact of road heterogeneity on the quality of predictions [17]. This paper contributes to this growing body of literature in a number of ways, including tests incorporating higher resolution imagery, exploration of the effectiveness of intercontinental transfer learning, and the implementation of a continuous measurement of road roughness."

Comment R2.2 *The 1st section can be split into introduction and related works.*
Answer to R2.2 Our first section has been reworked, following both this comment and comment 1.4. We believe the revised structure is much more effective, and thank the reviewers for their comments in this regard.

Comment R2.3 *Summarize the findings of the related works in the form of a table.*
Answer to R2.3 We have added a new table - Table 1 - which provides these details at the end of the reworked first section.

Comment R2.4 *Some of the recent works on CNN and image processing applied in several applications such as the following can be discussed in the paper: "Hand gesture classification using a novel CNN-crow search algorithm, A novel PCA–whale optimization-based deep neural network model for classification of tomato plant diseases using GPU, Image-Based malware classification using ensemble of CNN architectures (IMCEC)".*
Answer to R2.4 Thank you for highlighting these pieces. Citations for these three papers have been added to the first sentence of Related works.

Comment R2.5 *Compare the current work with recent state-of-the-art.*
Answer to R2.5 In addition to more explicit descriptions of our contributions in the revised section 1 and discussion, we have also added an additional two sentences to our discussion that directly contrasts our findings to past results:

"Contrasted to the in-situ data collection approach of Cadamuro et al. in Kenya [17] (see Table 1), our transfer-learning approach performed 4 percentage points better in our binary Nigeria case study. This suggests that transfer-learning approaches may be able to achieve similar levels of accuracy to approaches reliant on in-situ data collection."
